# Planning for Aging: Are We Making Progress?

**DOI:** 10.1093/geroni/igaf122.1693

**Published:** 2025-12-31

**Authors:** Mildred Warner, Xue Zhang

**Affiliations:** Cornell University, Ithaca, New York, United States; The Pennsylvania State University, University Park, Pennsylvania, United States

## Abstract

Did increased recognition of the need to address aging, prompted by the COVID-19 pandemic, and increased funding provided by the American Rescue Plan lead to more age-friendly action at the community level in the U.S.? We present data from two nationally representative cross-sectional surveys we conducted of U.S. municipalities with the International City/County Management Association in 2019 (N = 1290) and 2023 (N = 896). These surveys of city managers measure if U.S. communities include attention to the needs of older adults in their plans, built environment and services. We find only slight improvement in attention to aging in economic development and emergency plans, and no improvement in comprehensive or transportation plans. The built environment is harder to change in the short term, and we find little movement. We also find little improvement in the number of older adult-focused services at the community level. We discuss possible explanations for why municipal attention to aging overall was stagnant, such as the long-time course requisite for implementing municipal changes, societal ageism, and the overall centering of urban communities in the global age-friendly communities framework. We further discuss these findings in light of predominant frameworks for age-friendly communities, championed by high-level organizations such as AARP and the American Planning Association, suggesting that these frameworks have not yet materialized into widespread social change. Future research needs to give more attention to age-friendly initiatives in suburban and rural communities.

